# Identification of key genes governing diester alkaloid biosynthesis in different tissues of Aconitum kusnezoffii

**DOI:** 10.3389/fpls.2026.1793597

**Published:** 2026-04-24

**Authors:** Rihan Wu, Qiang Fu, Yahan Wu, Erdunhu E, Qieer Sa, Guihua Bao, Selinge Bai

**Affiliations:** 1 College of Mongolian Medicine, Inner Mongolia Minzu University, Tongliao, Inner Mongolia, China; 2 National Mongolian Medicine Preparation Center, Inner Mongolia International Mongolian Medicine Hospital, Hohhot, Inner Mongolia, China; 3 Key Laboratory of Mongolian Medicine R&D Engineering of Ministry of Education, Inner Mongolia Minzu University, Tongliao, Inner Mongolia, China

**Keywords:** BAHD acyltransferase family, different tissues of Aconitum kusnezoffii, high performance liquid chromatography, high-throughput sequencing, O-methyltransferase, transcriptome

## Abstract

**Introduction:**

Aconitum kusnezoffii is a valuable medicinal plant in Mongolian medicine, and diester alkaloids including aconitine (AC), hypaconitine (HA), and mesaconitine (MA) are its main active and toxic components. However, the tissue-specific distribution of these alkaloids and the key genes involved in their biosynthesis remain unclear. This study aimed to clarify the content differences of diester alkaloids in different tissues and identify related biosynthetic genes.

**Methods:**

The contents of AC, HA, and MA in cortex, phloem, and xylem of Aconitum kusnezoffii were determined by high-performance liquid chromatography (HPLC). Transcriptome sequencing was performed on the three tissues, and functional annotation was carried out using NR, Swiss-Prot, and Pfam databases. Candidate genes involved in alkaloid biosynthesis were screened with E−Value < 1e−10 and Identity ≥ 70%. Multiple sequence alignment and phylogenetic tree construction were performed using MEGA software to analyze the evolutionary relationships of OMT enzymes and BAHD acyltransferases.

**Results:**

HPLC results showed that the xylem had the highest content of diester alkaloids. Several candidate enzyme genes related to alkaloid biosynthesis were identified. The expression patterns of OMT and BAHD genes in different tissues were consistent with the trend of alkaloid accumulation. Phylogenetic analysis indicated that these enzymes were evolutionarily conserved.

**Discussion:**

This study clarified the tissue-specific accumulation pattern of diester alkaloids in Aconitum kusnezoffii and identified key biosynthetic genes. The findings reveal the evolutionary characteristics of related enzymes and provide a theoretical basis for further exploring the biosynthetic mechanism of diester alkaloids.

## 1 Introduction

Ranunculaceae perennial herb *Aconitum Kunsnezoffii Reichb*.is distributed in Inner Mongolia, Northeast China, Shanxi, Hebei, Mongolia, Korea, Russia and other places. Its root tuber is used as medicine called *Aconitum kusnezoffii* (Zhang et al., 2026). It has been recorded for a long time in Chinese and Mongolian medical classics. It is commonly used in rheumatoid arthritis, cold coagulation abdominal pain, fall injury and other diseases. Mongolian medicine believes that kusnezoff monkshood is warm, spicy, light and toxic (Wang J. et al., 2025). Modern pharmacological studies have confirmed that Aconiti Kusnezoffii Radix and its preparations have analgesic, anti-inflammatory, antihypertensive and cardiovascular regulation effects (Qasem et al., 2022; Singhuber et al., 2009).

**Figure 1:**
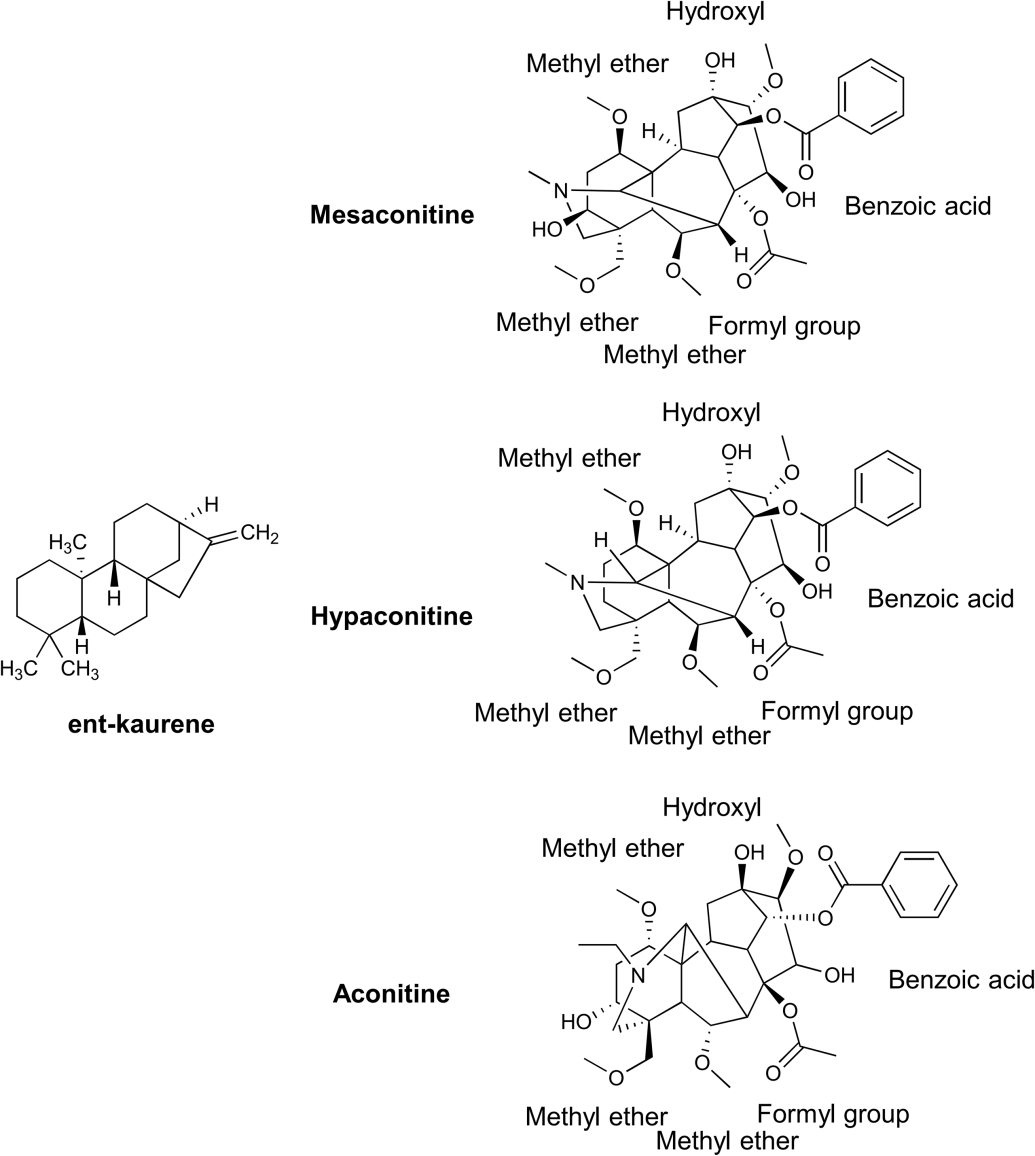
Chemical structures of the diester-diterpenoid alkaloids in *Aconitum kusnezoffii Radix*. [The chemical structures of aconitine (AC), hypaconitine (HA), and mesaconitine (MA) are presented, with key functional groups labeled, including benzoyloxy at C-14 and acetyloxy/hydroxyl at C-8. These diester-diterpenoid alkaloids are the main toxic and active components of Aconitum kusnezoffii Reichb].

The main active components of Aconiti Kusnezoffii Radix are diester alkaloids, including aconitine, hypaconitine and mesaconine (Jiang et al., 2021; Wang et al., 1998).Their mass fractions are 0.009% -0.012%, 0.035% -0.152% and 0.014% -0.190%, respectively (Sun et al., 2012), which are also the main toxic components. Studies have shown that the biosynthesis of diester alkaloids begins with the mevalonate pathway (MVA pathway) and the methyl erythritol phosphate pathway (MEP pathway), which together provide precursors to produce geranylgeranyl pyrophosphate (GGPP) (Yang et al., 2012). GGPP is catalyzed by type II terpenoid synthase (CPS) and type I terpenoid synthase (KSL) (Mao et al., 2021; Salehi et al., 2023) to generate ent-kaurene (Bai et al., 2022; Tian et al., 2023), which provides the core precursor for the subsequent formation of the unique C19-diterpenoid alkaloid skeleton of the bifingered alkaloids (Zhao et al., 2023). The precursor skeleton was hydroxylated by CYP450 enzyme (Luo et al., 2025), and part of the hydroxyl group was methylated by OMT (O-methyltransferase) to form methoxy (Wang T. et al., 2025; Wu et al., 2023), and then acylated by BAHD acyltransferase: the C14 position of the three was combined with benzoic acid to form benzoyloxy group, while the C8 position was different due to the catalytic specificity of the enzyme-the C8 hydroxyl group of aconitine and mesaconitine was acetylated to form acetoxy group, and the C8 position of hypaconitine only retained hydroxyl group (Shen et al., 2022; Xu et al., 2023; Zhao et al., 2023). As shown in **Figure 1**.

However, the toxicity source of *Aconitum kusnezoffii* is also diester alkaloids (such as aconitine, hypaconitine and mesaconitine), and toxicity control has become the primary issue in its clinical application (Wang et al., 2024). At present, toxicity control and quality evaluation are mostly based on the determination of whole root content (Chauhan et al., 2024; Punia et al., 2022), and there is a lack of systematic analysis of the accumulation rules of toxic components in different tissues (Liao et al., 2023), which makes it difficult to accurately control the toxicity during medication, which seriously restricts the clinical safety application. Therefore, it has become a key scientific issue to elucidate the synthesis and accumulation mechanism of diester alkaloids in different tissues of Aconitum kusnezoffii from the genetic level. Traditional chemical methods are difficult to systematically reveal the gene expression patterns in the synthesis pathway. Transcriptomics can comprehensively analyze the gene expression profiles of different tissues, and then mine the key enzyme genes involved in alkaloid synthesis and reveal their tissue-specific expression patterns (Chen et al., 2022; Xu et al., 2021).

In this study, transcriptomics and high performance liquid chromatography were integrated to compare the differences in the accumulation of diester alkaloids in the cortex, phloem and xylem of Aconiti Kusnezoffii Radix. The OMT and BAHD acyltransferase genes that may be related to synthesis were screened, and their functional potential was verified by evolution and expression analysis. The research results can provide a theoretical basis for elucidating the tissue-specific synthesis mechanism of the toxic components of Aconiti Kusnezoffii Radix, and also provide new ideas for improving the safety of its medication through gene regulation.

## 2 Materials and methods

### 2.1 Materials

The experimental medicinal materials were collected from Tejinhan Mountain, Zhalute Banner, Tongliao City, Inner Mongolia (116° 33 ′ 13 ′′ E, 45° 00 ′ 19 ′′ N, 1442 meters above sea level) in September 2020. Plants with good growth, no obvious pests and diseases and wilting leaves were selected, and the complete roots were dug and the surface soil was washed with clear water. After that, the roots were immersed in 75% ethanol solution for a short time to disinfect the surface, and washed with distilled water to remove residual ethanol. The root samples were placed on ice, and tissue separation was performed using a sterile scalpel: the outer epidermis (P), its inner phloem (R), and the central xylem (M) were removed in turn. After separation, each tissue was quickly put into liquid nitrogen for freezing. Each tissue type was repeated with 3 samples, and each sample was taken from three independent plants. A total of 9 groups of samples were obtained and immediately frozen in liquid nitrogen. The samples were stored at-80 °C for subsequent extraction experiments. It was identified as Aconitum kusnezoffii Reichb.by Professor Bao Guihua from the Mongolian Medicine Identification and Variety Resources Research Office of Inner Mongolia University for Nationalities.

### 2.2 Alkaloid determination method

#### 2.2.1 Extraction of aconitine

The dried cortex, phloem and xylem tissue powder 1.0 g (sieved through 80 mesh) were put into 100 mL plugged conical bottles, 3 mL ammonia test solution (analytical purity, Tianjin Damao Chemical Reagent Factory, China) was added, and 50 mL mixed solution of ethyl acetate-isopropanol (1: 1) (analytical purity, Tianjin Damao Chemical Reagent Factory, China) was added. Weighing, ultrasonic (power 300 W, frequency 40 kHz); kQ5200DE ultrasonic cleaner, Kunshan Ultrasonic Instruments Co., Ltd., China) water temperature below 25 °C treatment for 30 minutes, weighed after cooling, with ethyl acetate-isopropanol (1: 1) mixed solution to make up for the loss of weight, shake well, filter paper (11cm fast filter paper); the filtrate (25mL) was collected at 40 °C under reduced pressure and recovered to dryness (EYEL4 rotary evaporator, Shanghai Airang Instrument Co., Ltd., China). 3mL chloroform-isopropanol (1: 1) mixed solution was added, shaken, and filtered by filter paper (7cm fast filter paper). 1.25 mL filtrate was accurately measured and placed in a 5 mL measuring bottle. The mixed solution of chloroform-isopropanol (1: 1) was scaled and filtered with a 0.22 μm microporous filter (disposable organic filter, Tianjin Jinteng Experimental Equipment Co., Ltd., China) to obtain the test solution.

#### 2.2.2 Standard solution preparation

The standard aconitine (1.14 mg, Batch No.1170977-201607, National Institutes for Food and Drug Control, China), hypaconitine (2.01 mg, Batch No.110798-201609, National Institutes for Food and Drug Control, China), mesaconitine (2.25 mg, Batch No.110799-201608, National Institutes for Food and Drug Control, China) were accurately weighed and placed in 10 mL volumetric flasks. Trichloromethane-isopropanol (1: 1) mixed solution was added to the scale to obtain a single standard stock solution with a concentration of aconitine (0.114mg/mL), hypaconitine (0.201mg/mL), and mesaconitine (0.225mg/mL).

#### 2.2.3 HPLC chromatographic conditions

Chromatographic analysis was performed on a Waters RP18 column (4.6 mm × 250 mm, 5 μm, Waters, USA) using a high performance liquid chromatograph (SHIMADAZU LC-20A, Shimadzu Enterprise Management Co., Ltd., China); mobile phase: methanol (chromatographically pure, Merck, Germany) -0.1% triethylamine (analytically pure, Tianjin Damao Chemical Reagent Factory, China) (volume ratio 57: 43); detection wavelength: 235nm; column temperature: 35 °C; flow rate: 1.0mL/min; injection volume: 10 μL; elution method: isocratic elution.

#### 2.2.4 Content calculation and representation

The content of alkaloids was calculated by dry weight (mg/g dry weight, DW), and the formula was: content (mg/g) = (sample peak area/standard peak area) × standard concentration × dilution multiple/sample dry weight.

#### 2.2.5 Statistical analysis methods

Three biological replicates (n = 3) were set for each tissue, and each replicate was from an independent plant. One-way ANOVA was used to evaluate the differences between groups, and Duncan ‘s multiple comparison test was used for pairwise comparison between groups. The statistical significance level was set at P < 0.05. The data were expressed as mean ± standard deviation (SD).

### 2.3 Transcriptome method

#### 2.3.1 RNA extraction, sequencing and annotation

Total RNA was extracted from the samples using the TRIzol method. The concentration and purity of RNA after extraction were detected by Nanodrop2000, the integrity of RNA was detected by agarose gel electrophoresis, and the RIN value was determined by Agilent2100. The requirements of a single library were RNA (total) ≥ 1ug, concentration ≥ 35ng/μL, OD260/280 ≥ 1.8, OD260/230 ≥ 1.0. After qualified, the sequencing experiment used SMART-Seq _ V4UltraLowInputRNAKitforSequencing (micro) method for library construction. The original sequencing data was subjected to quality control filtering by fastp to obtain high-quality clean data. Trinity was used for *de novo* assembly, and TransRate and CD-HIT were used to optimize and de-redundant the assembly results. The assembly integrity was evaluated by BUSCO. The results showed that the proportion of complete BUSCO was 74.0%, including 68.9% of single copy and 5.1% of multiple copies. The assembled transcript sequences were aligned to six functional databases (NR, Swiss-Prot, Pfam, COG, GO, KEGG) by Diamond and HMMER software to obtain functional annotation information and protein domain annotation results, which provided the basis for subsequent expression and enrichment analysis.

#### 2.3.2 Core enzyme screening principles

When screening target enzyme genes from the results of transcriptome functional annotation, it is necessary to meet the following conditions: Blast alignment NR, Swiss-prot, Pfam database E-value < 1e-10; sequence identity (Identity) ≥ 70%; the annotation results clearly contain functional keywords such as ‘ OMT ‘, ‘ BAHD acyltransferase ‘, ‘ demethylase ‘, etc., to ensure the functional credibility of the screened genes.

#### 2.3.3 Conservative motif analysis

In order to further evaluate the functional credibility of candidate genes, the MEME online tool (version 5.4.1) was used to identify the conserved motifs of their encoded proteins. The parameter is set as follows: the width of the motif is 6–50 amino acids, the maximum number of motifs found is 10, and the remaining parameters are default. The identified motifs were compared with the OMT and BAHD proteins that had been functionally verified in the NCBI non-redundant protein database (Nr), and the similarity was evaluated by E-value to determine whether the candidate genes contained typical functional domains.

#### 2.3.4 Evolutionary analysis of key enzymes and pathway visualization

Based on the target sequence in the transcript, the Neighbor-Joining method of ‘ MEGA software was used to construct the phylogenetic tree by Bootstrap repeating 1000 times to clarify the genetic relationship between the target species (Aconitum kusnezoffii Reichb.) and other species. The genetic relationship between OMT, BAHD and homologous proteins of different families and genera was clarified by cluster analysis, and then the conservatism of their functions and potential differentiation characteristics were inferred, and the false positives caused by random evolution were excluded. Based on the KEGG pathway annotation and the published literature on the synthesis of diterpenoid alkaloids in Aconitum, the putative biosynthetic pathways of aconitine (AC), hypaconitine (HA) and mesaconitine (MA) were integrated and confirmed. Adobe Illustrator 2023 software was used to draw a schematic diagram of the pathway. The key precursors, intermediates, final products and key enzymes involved in each step of the catalytic reaction were clearly marked in the diagram, so as to intuitively show the key links in the synthesis of diester alkaloids from Radix Aconiti Kusnezoffii and the potential action sites of the genes screened in this study.

### 2.4 Data availability

The data presented in the study are deposited in the Genome Sequence Archive (GSA) repository, accession number PRJCA061128. The data will be made publicly available immediately upon publication of this article.

## 3 Experimental results

### 3.1 Distribution characteristics of diester alkaloids in different tissues of Aconitum kusnezoffii

**Figure 2:**
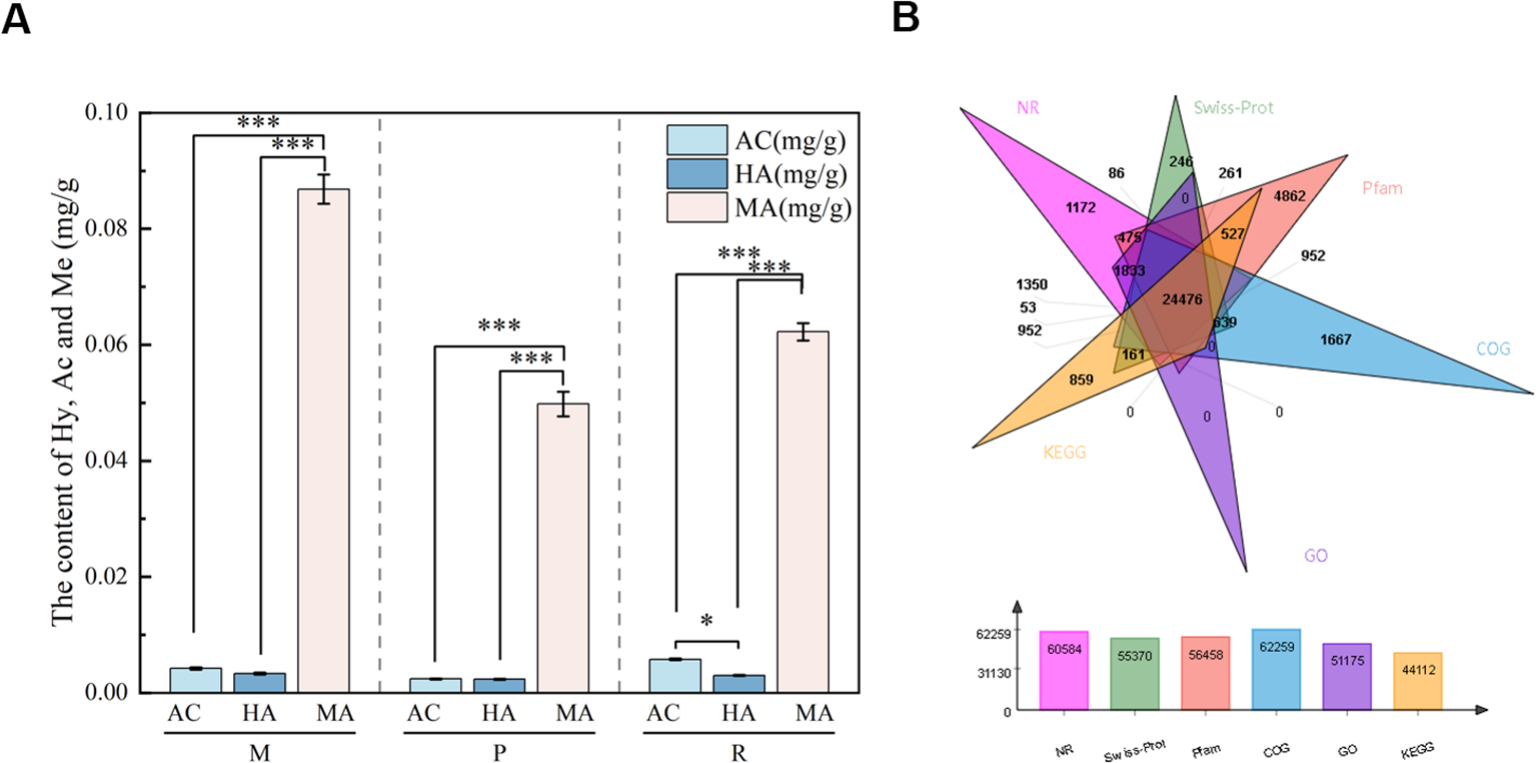
**(A)** Contents of AC, HA, and MA in different tissues of *Aconitum kusnezoffi*i Radix. [The contents of aconitine (AC), hypaconitine (HA), and mesaconitine (MA) in cortex (P), phloem (R), and xylem (M) tissues were quantified by high-performance liquid chromatography (HPLC). Values represent means of three biological replicates, and error bars indicate standard deviations (SD).]Data are expressed as mean ± SD (n=3). Different numbers of * indicate significant differences among tissues (* = P < 0.05, *** = P < 0.001, One-way ANOVA with Duncan’s test). **(B)** Functional annotation results of Unigenes from *Aconitum kusnezoffii* Radix. [The charts show the distribution of Unigenes annotated in six major databases (NR, Swiss-Prot, Pfam,COG, GO, KEGG) following transcriptome sequencing of three tissue types (cortex, phloem, xylem) of *Aconitum kusnezoffii* Radix].

The contents of three diester alkaloids in cortex (P), phloem (R) and xylem (M) of Aconiti Kusnezoffii Radix were determined by HPLC,As shown in **Figure 2A**.

### 3.2 Core enzyme gene screening and expression correlation

The transcriptome analysis of 9 samples of cortex (P1, P2, P3), xylem (M1, M2, M3) and phloem (R1, R2, R3) of Aconitum kusnezoffii was completed, and a total of 61.18 Gb Clean Data was obtained. The Clean Data of each sample was greater than 6.2 Gb, and the percentage of Q30 bases was greater than 91.98%. Clean Data *de novo* assembly was performed on all samples using Trinity software, and the assembly results were optimized and evaluated. The number of Unigenes obtained was 153213, the number of Transcriptions was 215430, the average length was 563.49 bp, and the N50 length was 808 bp. The high quality sequence of Q20 in 9 samples accounted for more than 97.03%, and the vast majority of them were more than 45.02% of the total bases of GC content. The data show that the sequence quality obtained by transcriptome sequencing in different tissues of *Aconiti Kusnezoffii Radix* is good, and the data is extensive and the assembly data can meet the conditions of the original data. BUSCO integrity assessment showed that the assembly integrity of Unigene was 74.0%, of which single-copy genes accounted for 68.9% and multi-copy genes only accounted for 5.1%, indicating that the assembly results were reliable and the redundancy removal effect was good, which could be used for subsequent functional annotation and gene mining. The assembled 153213 Unigenes were compared with Blast and six public databases. A total of 60584,55370,56458,62259,51175 and 44112 Unigenes were annotated in NR, Swiss-Prot, Pfam, COG, GO and KEGG databases, respectively. The annotation results of each database are shown in **Figure 2B**.

**Figure 3:**
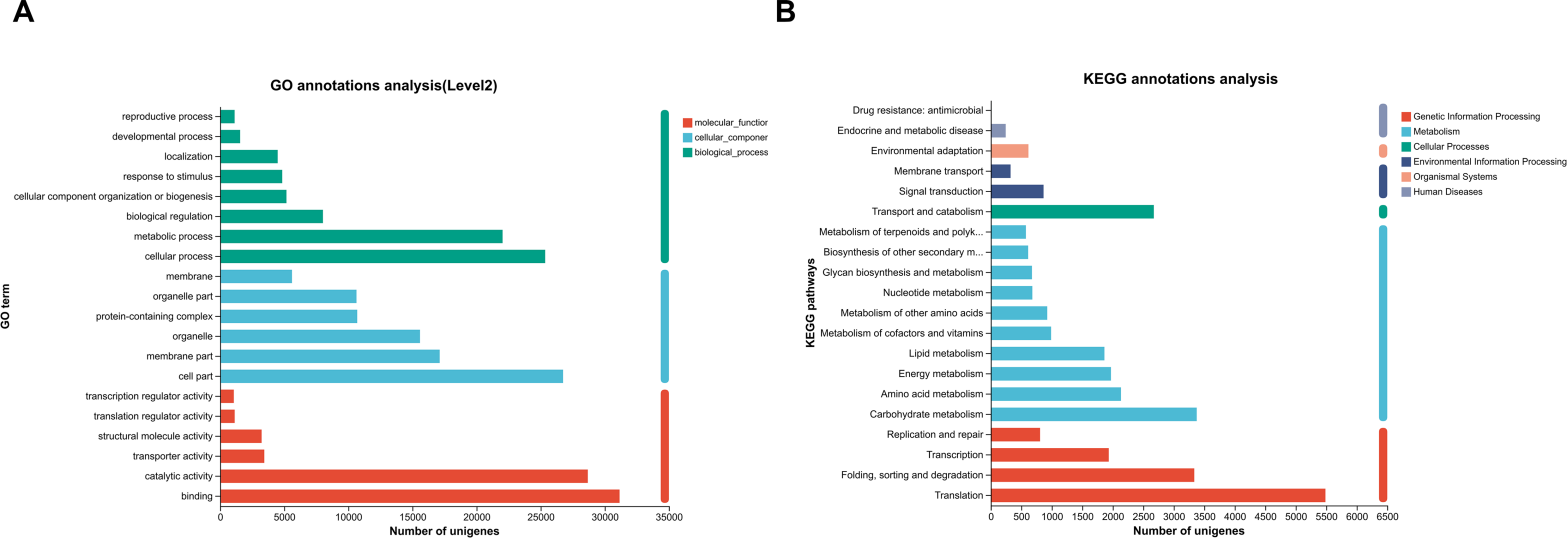
**(A)** GO classification map of Unigenes from *Aconitum kusnezoffii Radix*. [Unigenes were categorized into three main ontologies: Biological Process (BP), Cellular Component (CC), and Molecular Function (MF). The percentages indicate the proportion of genes assigned to each subcategory.] **(B)** KEGG classification map of Unigenes from Aconitum kusnezoffii Radix. [Unigenes mapped to KEGG metabolic pathways were grouped into six major categories, with the number and percentage of genes in each category displayed].

In the transcriptome functional annotation analysis, a total of 51,702 GO annotations of Unigenes were obtained. These genes were divided into three categories: Biological Process (BP), Cellular Component (CC) and Molecular Function (MF), as shown in **Figure 3A**. Among them, the cell component (CC) annotation accounted for the highest proportion (38.27%), mainly involving ‘ cell ‘ and ‘ cell membrane ‘ related functions; biological process (BP) accounted for 31.65%, mainly ‘ cellular process ‘ and ‘ metabolic process ‘. Molecular function (MF) accounted for 30.08%, with ‘ catalytic activity ‘ and ‘ binding activity ‘ as the main functions. It is worth noting that the ‘ enzyme catalytic activity ‘ related genes closely related to the synthesis of diester alkaloids account for a relatively high proportion in the MF category, which provides a clear direction for the subsequent screening of key functional genes. A total of 44,568 Unigenes were mapped to 135 metabolic pathways by KEGG pathway analysis, and they were classified into 6 major categories. The results are shown in **Figure 3B**. Among them, ‘ Metabolic pathways ‘ covered the largest number of genes (17,632, accounting for 39.6%), while ‘ Biosynthesis of other secondary metabolites ‘ category annotated 1,101 genes, including ‘ diterpenoid alkaloid biosynthesis ‘, ‘ terpenoid skeleton biosynthesis ‘ and other pathways closely related to the target product. The results further focused on the metabolic network level, providing a reliable candidate target and functional background for the systematic mining of structural genes and regulatory factors involved in the synthesis of diester alkaloids.

#### 3.2.1 Identification of OMT transferase and BAHD acyltransferase

From the results of transcriptome functional annotation, according to E-value < 1e-10, Identity ≥ 70% and functional keyword matching, candidate genes that may be involved in the synthesis of diester alkaloids were screened, including the OMT enzyme gene TRINITY _ DN39644 _ c1 _ g1. And BAHD acyltransferase gene TRINITY _ DN51911 _ c0 _ g3, TRINITY _ DN120237 _ c0 _ g1, TRINITY _ DN781 _ c3 _ g2, TRINITY _ DN21886 _ c0 _ g1.

#### 3.2.2 Expression correlation

**Figure 4:**
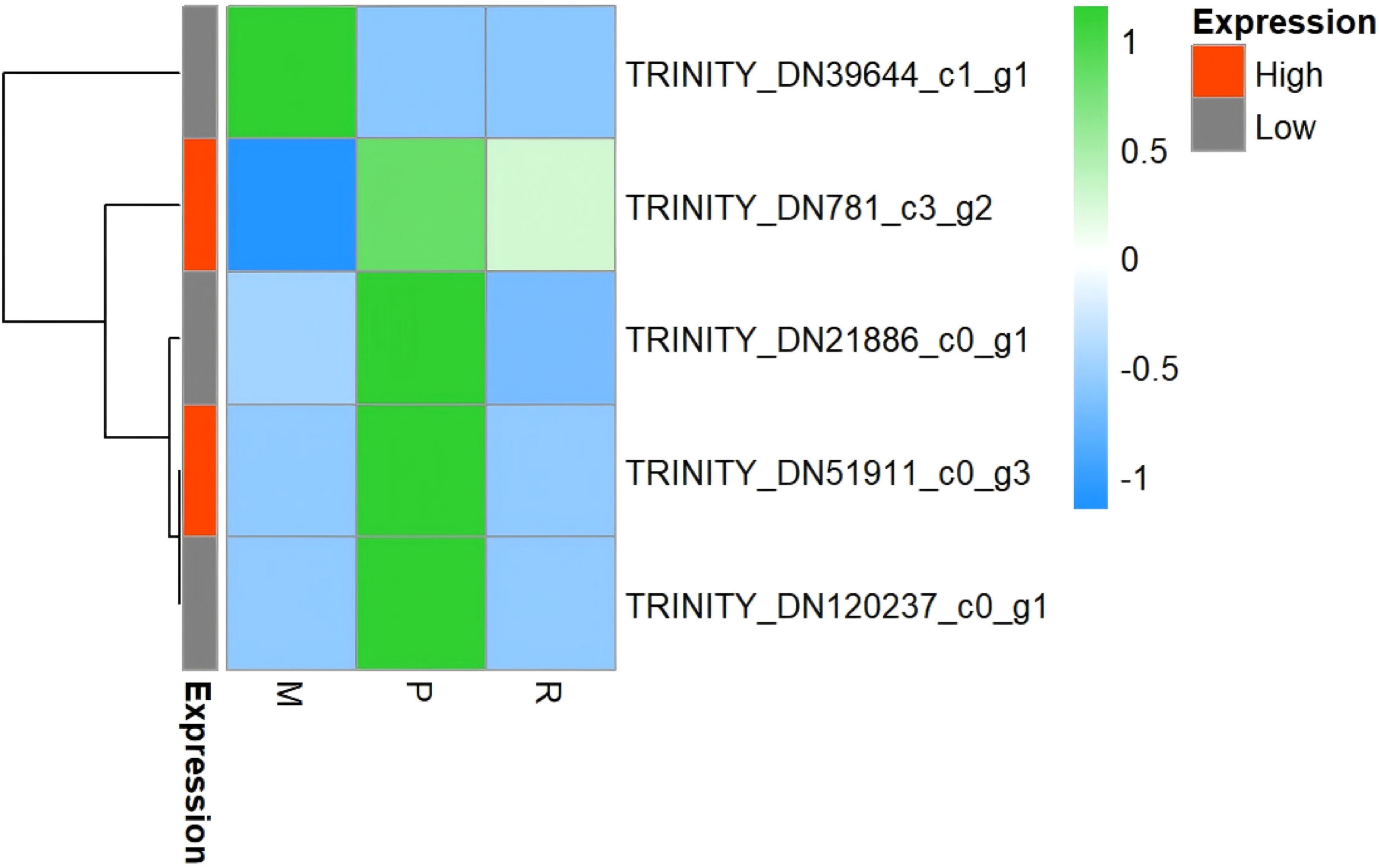
Heatmap of expression levels of candidate key enzyme genes in different tissues of *Aconitum kusnezoffii Radix*. [The heatmap shows expression profiles of one O-methyltransferase (OMT) gene and four BAHD acyltransferase genes in cortex (P), phloem (R), and xylem (M) tissues. Color intensity indicates normalized expression levels, with red representing high expression and blue representing low expression].

In order to clarify the correlation between alkaloid content and key enzyme gene expression in different parts, the heat map of key enzyme gene expression was drawn, as shown in **Figure 4**. The heat map showed that the gene expression showed obvious tissue specificity: TRINITY _ DN39644 _ c1 _ g1 (OMT) and TRINITY _ DN21886 _ c0 _ g1 (BAHD) were highly expressed in xylem (M), and lowly expressed in cortex (P) and phloem (R). Combined with the distribution characteristics of aconitine, hypaconitine and mesaconitine in different tissues of Aconitum kusnezoffii, it can be seen that the high content part of MA alkaloid is xylem (M), which is consistent with the accumulation trend of high expression parts of TRINITY _ DN39644 _ c1 _ g1 and TRINITY _ DN21886 _ c0 _ g1. This result suggests that xylem is not only the main accumulation part of MA, but also the main expression part of candidate genes, which further supports the reliability of these two genes as candidate genes involved in MA biosynthesis. Based on this, it is speculated that xylem may be the main site for MA synthesis or accumulation, but its exact synthesis site and gene function still need to be verified by subsequent experiments.

#### 3.2.3 Conserved motif analysis of candidate genes

**Figure 5:**
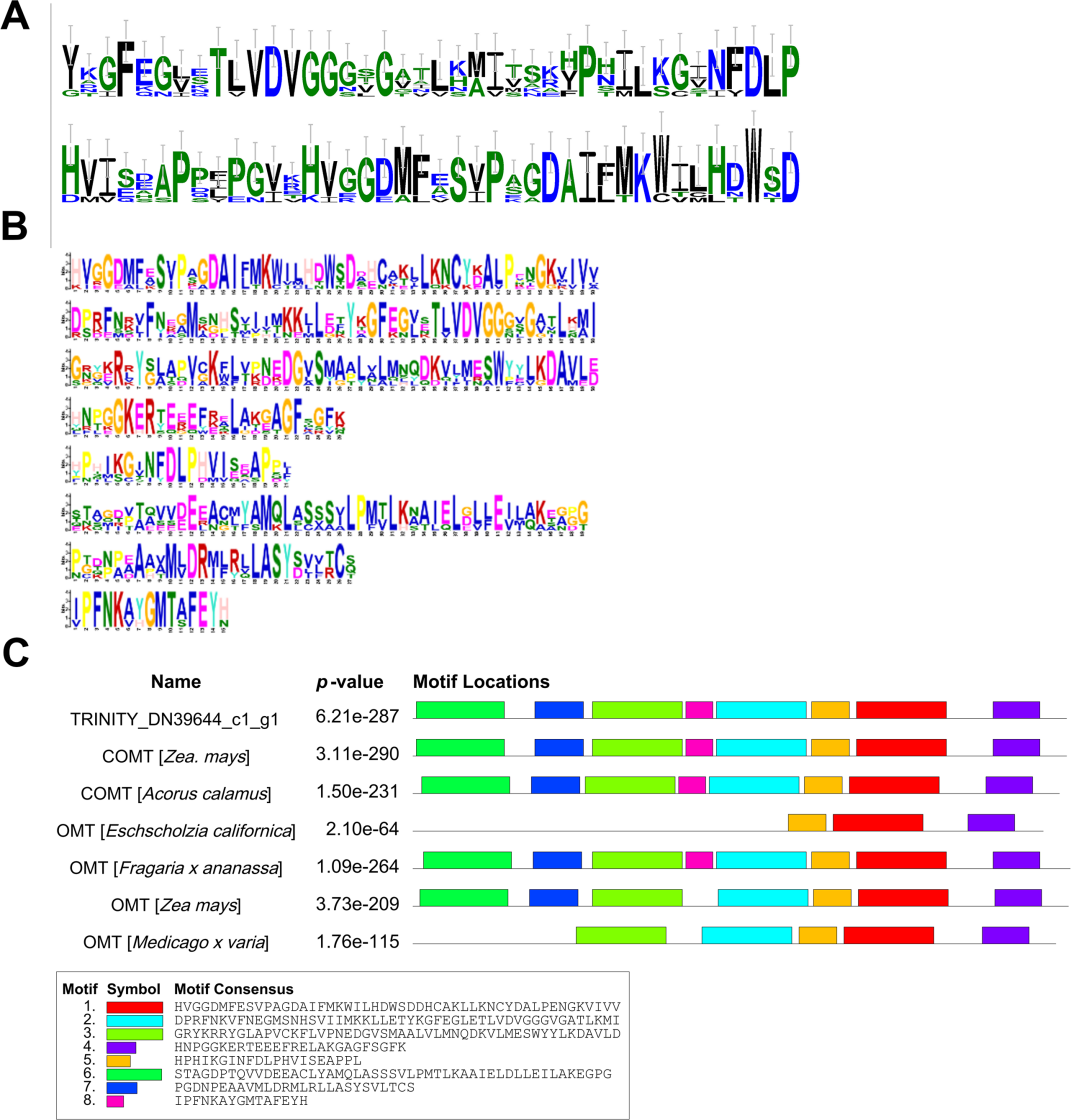
Conserved motif analysis of O-methyltransferase (OMT) candidate genes. **(A)** Conserved motifs identified in OMT candidate genes; **(B)** OMT candidate genes and multiple conserved motifs identified in known functional genes; **(C)** The comparison results of OMT candidate genes with other plants have been functionally verified.

In order to evaluate the functional reliability of candidate genes, MEME online tool was used to identify the conserved motifs of their encoded proteins, and sequence alignment was performed with known functional enzymes from other species. The results showed that the protein encoded by the candidate OMT gene (TRINITY _ DN39644 _ c1 _ g1) contained several typical plant OMT conserved motifs, such as ‘ BYGFEGVTLVDVGGCGLK ‘ and ‘ BHVISAPPEPSYGKVGGDMFSYPADAIEMKLHLHPD ‘ as shown in **Figure 5**. These motifs are spatially corresponding to the S-adenosylmethionine binding domain and substrate binding pocket, respectively. They are highly similar to functional OMTs such as CCOMT from Zea mays and COMT from Eschscholzia californica, suggesting that they may have methyltransferase activity.

**Figure 6:**
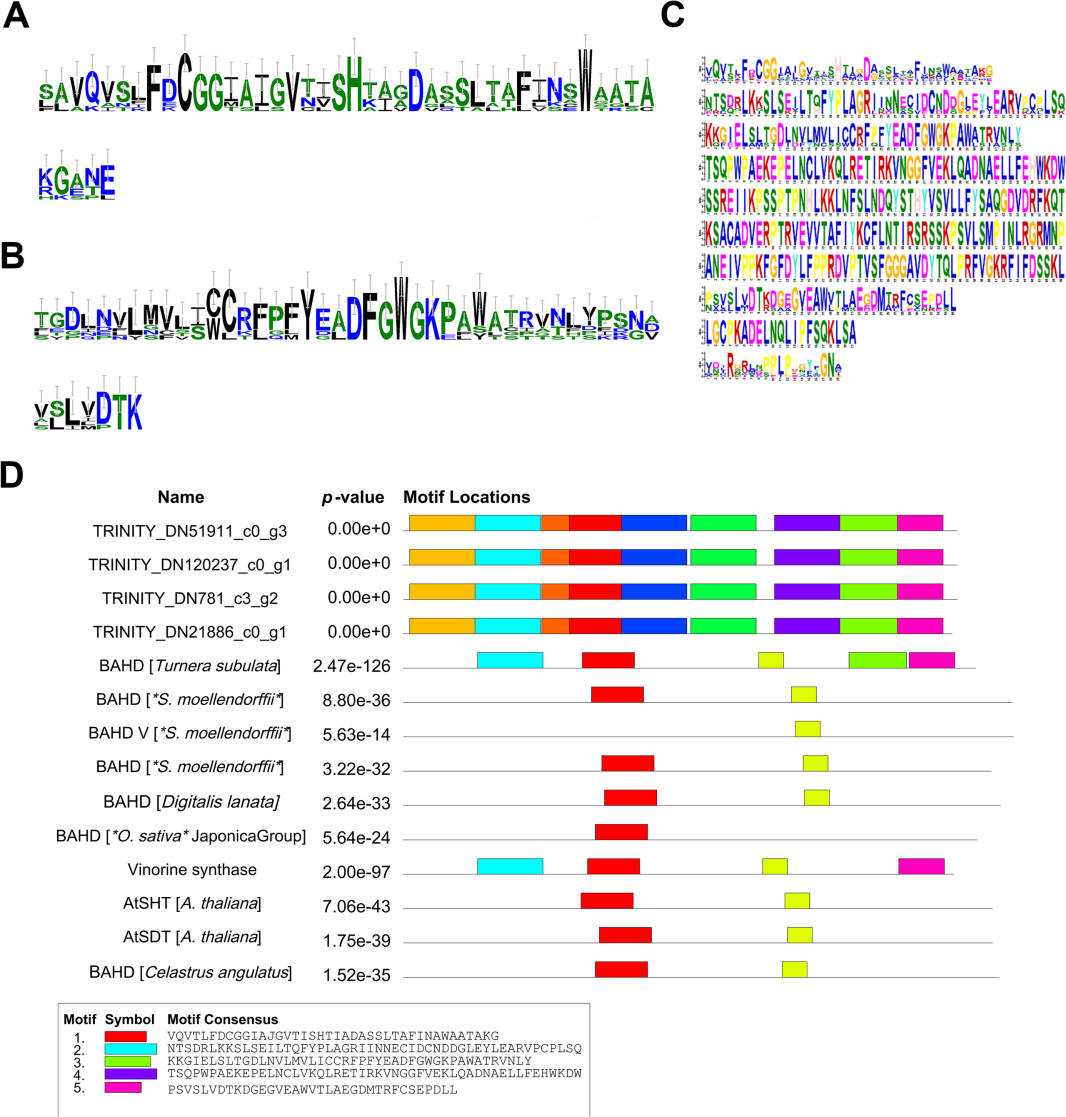
Conserved motif analysis of BAHD acyltransferase candidate genes. **(A)** Sequence diagram of the characteristic motif ‘ HXXXD ‘ of BAHD family; **(B)** Sequence diagram of BAHD family characteristic motif ‘DFGWG ‘; **(C)** Detailed sequences of four BAHD candidate genes and multiple conserved motifs identified in known functional genes; **(D)** Candidate genes have been functionally verified with other plants BAHD acyltransferase alignment results.

At the same time, the motif analysis of four BAHD acyltransferase candidate genes (TRINITY _ DN51911 _ c0 _ g3, TRINITY _ DN120237 _ c0 _ g1, TRINITY _ DN781 _ c3 _ g2, TRINITY _ DN21886 _ c0 _ g1) showed that they all contained characteristic conserved domains of the BAHD family, such as ‘ HXXXD ‘ and ‘ DFGWG ‘ motifs as shown in **Figure 6**. Among them, the ‘ HXXXD ‘ motif is a key catalytic site for acyl transfer reaction, which is responsible for coenzyme A binding and acyl transfer, while the ‘ DFGWG ‘ motif is essential for maintaining the stability of protein structure. The alignment results with known functional enzymes showed that these motifs were highly consistent with BAHD enzymes that have been reported to be involved in the acylation of terpenoids and alkaloids (such as functional genes in Arabidopsis thaliana and Catharanthus roseus), further supporting their potential functions in the C14 benzoylation or C8 acetylation of diester alkaloids.

### 3.3 Evolutionary characteristics and synthetic pathways of key enzymes

#### 3.3.1 Phylogenetic tree results

**Figure 7:**
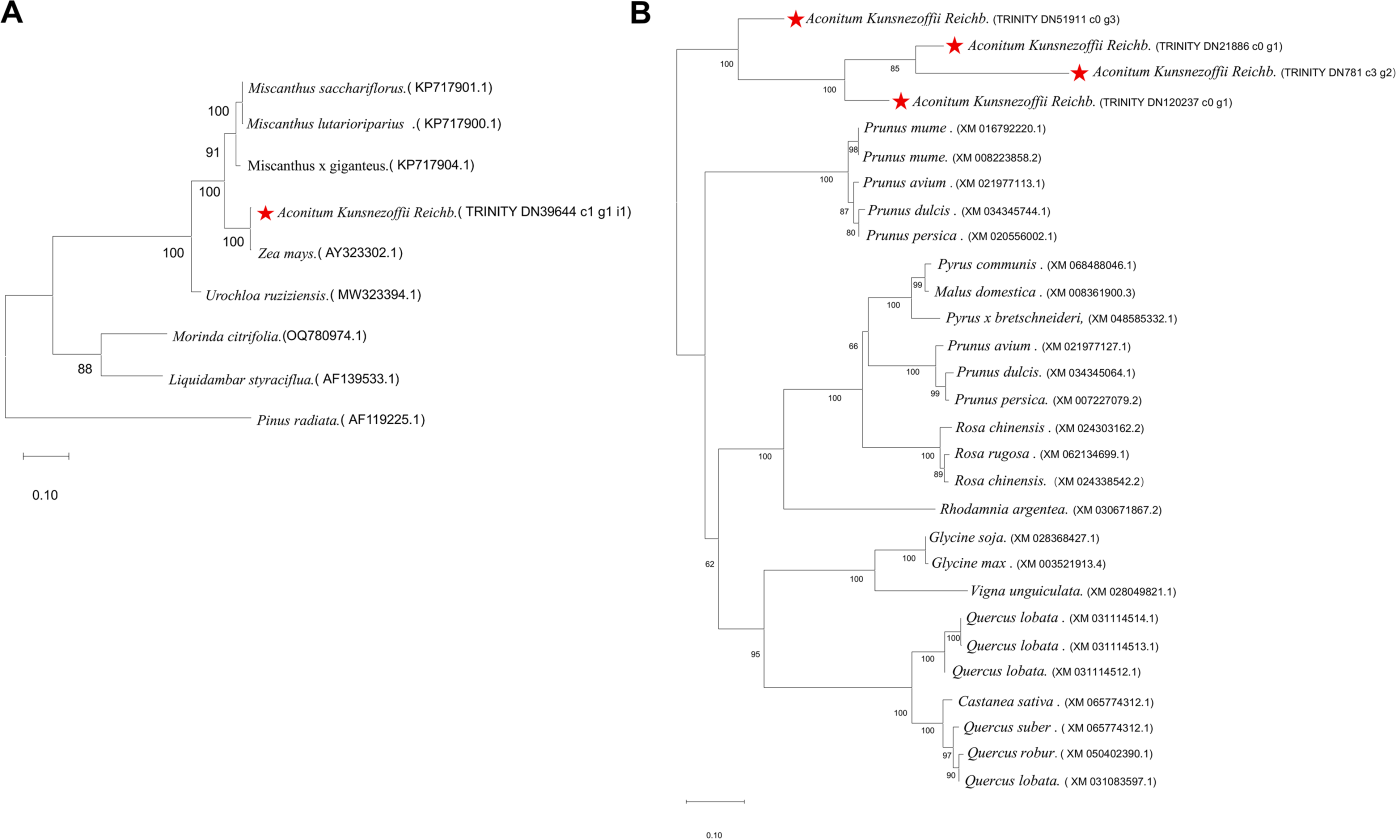
**(A)** Phylogenetic tree of O-methyltransferases (OMTs) from *Aconitum kusnezoffii Radix*. and other plant species. [The tree was constructed using the Neighbor-Joining method with 1000 bootstrap replicates. Branches are colored according to species groups, and bootstrap values >70% are shown]. **(B)** Phylogenetic analysis of BAHD acyltransferases from *Aconitum kusnezoffii Radix*. and related species. [The tree includes sequences from Rosaceae, Leguminosae, Fagaceae, and other plant families. The clade containing A. kusnezoffii sequences is highlighted, with bootstrap support values indicated].

In order to explore the evolutionary relationship between OMT transferase, BAHD acyltransferase and related proteins in different species of Aconitum kusnezoffii, MEGA software was used to analyze 9 species of OMT transferase, such as Miscanthus, corn and so on. The BAHD acyltransferases of 24 species in Rosaceae (Prunus, Rosa, Malus, Pyrus), Leguminosae (Glycine, Vigna) and Fagaceae (Quercus, Castanea) were compared by multiple sequence alignment. The results are as follows: The phylogenetic tree related to OMT transferase of Aconitum kusnezoffii, as shown in **Figure 7A**. The 9 proteins were divided into 3 branches: the first branch (7 members): Miscanthus subgroup: Miscanthus sacchariflorus had the closest relationship with M.lutarioriparius, and then clustered with M.x giganteus; mixed subgroup: Aconitum kusnezoffii (TRINITY DN39644) had the closest relationship with maize (AY323302.1), and then clustered with lemma grass. Second branch: Morinda officinalis and Liquidambar formosana clustering. The third branch: radial pine is an independent branch. The BAHD acyltransferase-related phylogenetic tree of Aconiti Kusnezoffii Radix is shown in **Figure 7B**. The sequences (TRINITY DN51911, DN21886, DN781, DN120237) of Aconiti kusnezoffii Radix were clustered into an independent branch with 100% bootstrap support, reflecting the close evolutionary relationship between homologous proteins of Aconiti kusnezoffii Radix. The evolutionary relationship with homologous proteins of Achillea and Andrographis paniculata was relatively close; the BAHD proteins of oil palm and rhododendron showed branch specificity and had a long evolutionary distance from other species.

#### 3.3.2 Synthetic pathway

**Figure 8:**
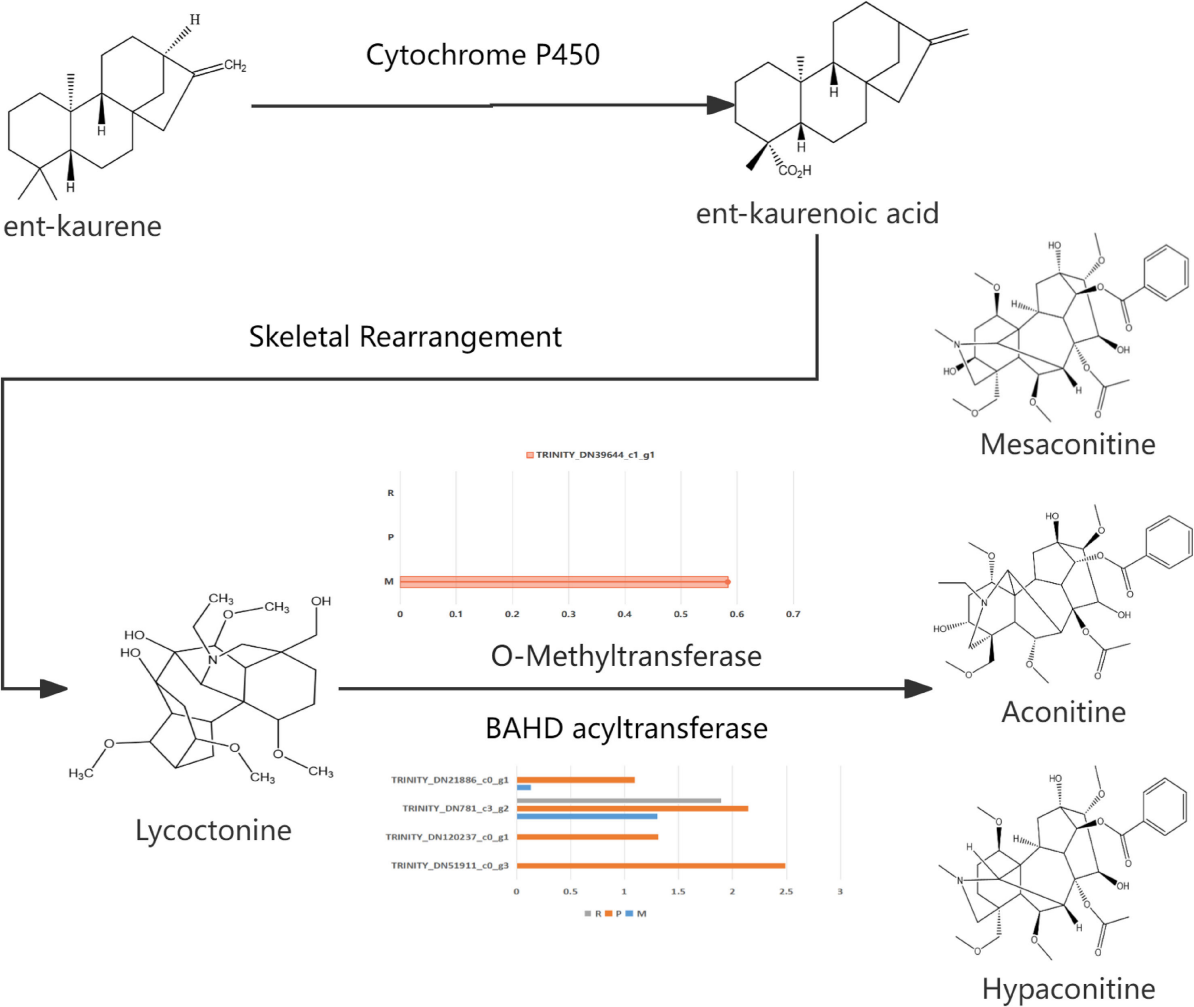
Proposed biosynthetic pathway of diester-diterpenoid alkaloids in *Aconitum kusnezoffii Radix*. [This pathway illustrates key intermediates, final products (aconitine, AC; hypaconitine, HA; mesaconitine, MA), and candidate enzymes (O-methyltransferase,; BAHD acyltransferase) involved in the conversion from ent-kaurene to diester-diterpenoid alkaloids, based on KEGG annotation and published literature].

Based on the KEGG annotation and related literature, the biosynthetic pathway map of the diester alkaloids of Aconitum kusnezoffii was drawn, as shown in **Figure 8**. This pathway covers the key catalytic steps and related enzymes from the precursor compound ent-kaurene to the final products AC, HA, and MA.

## 4 Discussion

According to the results of HPLC, the three diester alkaloids showed a gradient distribution of xylem > phloem > cortex in different tissues of Aconitum kusnezoffii. However, the traditional medicinal use of Aconitum kusnezoffii is mostly used as a whole root, which may lead to the mixing of ineffective tissues (such as cortex) and the low utilization rate of medicinal ingredients. Through the annotation of six major databases, it could support the screening of key enzyme genes and lay the foundation for subsequent analysis. However, there are still some shortcomings: first, the sample is limited. The samples are only collected from wild Aconitum carmichaelii in a single production area and a single phenological period. The geographical and temporal representation is insufficient, and the universality of the results is poor; secondly, the candidate genes (such as OMT, BAHD) have not been verified by *in vitro* expression or gene silencing, and the key gene attributes are only speculated.

Through analysis, several key enzyme genes that may be involved in the synthesis of diester alkaloids were successfully screened and identified from the cortex, phloem and xylem of Aconiti Kusnezoffii Radix, including one O-methyltransferase gene (OMT) and four BAHD acyltransferase genes. These genes were highly expressed in xylem and showed spatial consistency with the high accumulation site of mesaconitine (MA), suggesting that they may be candidate genes involved in MA biosynthesis, suggesting that they may play an important role in the biosynthesis of MA. In the known diterpenoid alkaloid synthesis pathway, OMT is responsible for catalyzing the methylation reaction of hydroxyl groups to form a methoxy structure, which is one of the key structural modification steps in aconitine, hypaconitine and mesaconitine. The OMT gene (TRINITY _ DN39644 _ c1 _ g1) identified in this study had the highest expression in xylem, which was consistent with the trend of MA accumulation. Phylogenetic tree analysis showed that the gene was close to the OMT cluster of gramineous plants such as maize (Zea mays), suggesting that it may have certain functional conservation in the catalytic mechanism. Combined with previous studies, plant OMT is often involved in the methylation modification of secondary metabolites, such as the synthesis of flavonoids and alkaloids. Therefore, the gene is likely to be responsible for catalyzing the methylation of the hydroxyl group of the precursor of diester alkaloids in Aconitum kusnezoffii and promoting the formation of MA.

It is worth noting that conserved motif analysis provides structural biological evidence for the functional credibility of candidate genes. The protein encoded by the OMT candidate gene (TRINITY _ DN39644 _ c1 _ g1) in this study contains typical plant OMT conserved motifs, which are highly similar to functionally verified OMTs such as maize (Zea mays) CCOMT and California poppy (Eschscholzia californica) COMT (E-value of 10 ^ -64 ~ 10 ^ -290). These motifs correspond to the S-adenosylmethionine binding domain and the substrate binding pocket, respectively. The four BAHD candidate genes all contain the ‘ HXXXD ‘ and ‘ DFGWG ‘ motifs unique to the BAHD family, which are completely consistent with the BAHD enzyme characteristics that have been reported to be involved in the acylation of terpenoids and alkaloids in Arabidopsis thaliana, Catharanthus roseus and other species. This result further supports the potential function of these candidate genes involved in the synthesis of diester alkaloids from the protein structure level. BAHD acyltransferases are widely involved in acylation reactions in plant secondary metabolism, especially in the modification of terpenoids and alkaloids. In this study, four BAHD acyltransferase genes (such as TRINITY _ DN51911 _ c0 _ g3, etc.) were identified, which were highly expressed in xylem and positively correlated with MA accumulation. It is known that BAHD enzyme is responsible for catalyzing C14 benzoylation and C8 acetylation (such as aconitine and mesaconitine), or retaining C8 hydroxyl (such as hypaconitine) in the synthesis of aconitine alkaloids. Therefore, these BAHD candidate genes may be involved in the C14 benzoylation (formation of benzoyloxy) and C8 acetylation or hydroxyl retention reaction, respectively, to determine the structural differences of the final products of AC, HA and MA.

## 5 Conclusion

In this study, HPLC quantitative analysis and transcriptome sequencing technology were integrated to systematically compare the accumulation differences of three diester alkaloids (aconitine, hypaconitine and mesaconitine) in cortex, phloem and xylem of Aconiti Kusnezoffii Radix. Based on transcriptome data, the key enzyme genes that may be involved in its synthesis were screened and identified, and the functional potential of these genes was further verified by evolutionary analysis and expression correlation. The diester alkaloids of Aconiti Kusnezoffii Radix have obvious tissue-specific distribution. The content of MA in xylem was the highest, followed by phloem, and the cortex was the lowest, while the content of AC and HA in each tissue was low and the difference was not significant. Several potential key enzyme genes were screened and identified: including one OMT gene and four BAHD acyltransferase genes, which were highly expressed in xylem and have spatial consistency with the high accumulation sites of MA, suggesting that they may be candidate genes involved in MA biosynthesis, suggesting that these genes may be involved in the biosynthesis of MA. Phylogenetic tree analysis revealed the conservation and differentiation characteristics of key enzymes. The OMT of Aconiti Kusnezoffii Radix was clustered with OMT of Miscanthus, maize and other species, while BAHD acyltransferase was clustered with Gramineae and other species, reflecting that its function was conservative and group-specific in the evolutionary process.

## Data Availability

The authors acknowledge that the data presented in this study must be deposited and made publicly available in an acceptable repository, prior to publication. Frontiers cannot accept a manuscript that does not adhere to our open data policies.

## References

[B1] Bai S. Sartagnuud S. Wang T. Bao G. Bao S. Ao W. (2022). De nove transcriptome sequencing identifies genes involved in aconitine-type alkaloids biosynthesis in Aconitum kusnezoffii Reichb. Pharmacol. Res. Mod. Chin. Med. 2, 100063. doi: 10.1016/j.prmcm.2022.100063. PMID: 41930316

[B2] Chauhan J. Kant Purohit V. Sharma U. (2024). Metabolomics integration with chemometrics for the quality assessment: A case study with commercially important Himalayan medicinal plant Aconitum heterophyllum Wall. Microchem. J. 199, 110129. doi: 10.1016/j.microc.2024.110129. PMID: 41930316

[B3] Chen L. Tian M. Jin B. Yin B. Chen T. Guo J. . (2022). Integrating metabolomics and transcriptomics to unveil atisine biosynthesis in Aconitum gymnandrum Maxim. Int. J. Mol. Sci. 23, 13463. doi: 10.3390/ijms232113463. PMID: 36362268 PMC9655601

[B4] Jiang Z. Guo H. Hu Y. Zhou L. Deng C. Nan Z. . (2021). Classification of diterpenoid alkaloids from Aconitum kusnezoffii Reichb. by liquid chromatography‐tandem mass spectrometry‐based on molecular networking. J. Sep. Sci. 45, 739–751. doi: 10.1002/jssc.202100651. PMID: 34865311

[B5] Liao H. Quan H. Huang B. Ji H. Zhang T. Chen J. . (2023). Integrated transcriptomic and metabolomic analysis reveals the molecular basis of tissue-specific accumulation of bioactive steroidal alkaloids in Fritillaria unibracteata. Phytochemistry 214, 113831. doi: 10.1016/j.phytochem.2023.113831. PMID: 37598994

[B6] Luo F. Zhou Q. Chen F. Liu X. Chiu T.-Y. Zhu G.-Y. . (2025). Divergent multifunctional P450s-empowered biosynthesis of bioactive tripterifordin and cryptic atiserenoids in Aconitum implies convergent evolution. Nat. Commun. 16, 5857. doi: 10.1038/s41467-025-61188-0. PMID: 40595667 PMC12219214

[B7] Mao L. Jin B. Chen L. Tian M. Ma R. Yin B. . (2021). Functional identification of the terpene synthase family involved in diterpenoid alkaloids biosynthesis in Aconitum carmichaelii. Acta Pharm. Sin. B. 11, 3310–3321. doi: 10.1016/j.apsb.2021.04.008. PMID: 34729318 PMC8546855

[B8] Punia A. Joshi R. Kumar R. (2022). Identification and quantification of eight alkaloids in Aconitum heterophyllum using UHPLC‐DAD‐QTOF‐IMS: A valuable tool for quality control. Phytochem. Anal. 33, 1121–1134. doi: 10.1002/pca.3164. PMID: 35794832

[B9] Qasem A. M. A. Zeng Z. Rowan M. G. Blagbrough I. S. (2022). Norditerpenoid alkaloids from Aconitum and Delphinium: structural relevance in medicine, toxicology, and metabolism. Nat. Prod. Rep. 39, 460–473. doi: 10.1039/d1np00029b. PMID: 34636385

[B10] Salehi A. Ghanadian M. Zolfaghari B. Jassbi A. R. Fattahian M. Reisi P. . (2023). Neuropharmacological potential of diterpenoid alkaloids. Pharmaceuticals 16, 747. doi: 10.3390/ph16050747. PMID: 37242531 PMC10223254

[B11] Shen Y. Liang W.-J. Shi Y.-N. Kennelly E. J. Zhao D.-K. (2022). Correction: Structural diversity, bioactivities, and biosynthesis of natural diterpenoid alkaloids. Nat. Prod. Rep. 39, 2338–2340. doi: 10.1039/D2NP90035A. PMID: 36458680

[B12] Singhuber J. Zhu M. Prinz S. Kopp B. (2009). Aconitum in traditional Chinese medicine—A valuable drug or an unpredictable risk? J. Ethnopharmacol. 126, 18–30. doi: 10.1016/j.jep.2009.07.031. PMID: 19651200

[B13] Sun A. Gao B. Ding X. Huang C.-M. But P. P.-H. (2012). Quantitative and qualitative analysis of Aconitum alkaloids in raw and processed Chuanwu and Caowu by HPLC in combination with automated analytical system and ESI/MS/MS. J. Anal. Methods Chem. 2012, 1–7. doi: 10.1155/2012/936131. PMID: 22567575 PMC3335326

[B14] Tian M. Jin B. Chen L. Ma R. Ma Q. Li X. . (2023). Functional diversity of diterpene synthases in Aconitum plants. Plant Physiol. Biochem. 202, 107968. doi: 10.1016/j.plaphy.2023.107968. PMID: 37619270

[B15] Wang J. Bao L. Zhao Y. Song L. Zu W. Wang J. . (2025). Mongolian medicine theory-based multidimensional evaluation of toxicity mitigation in Hezi-processed Caowu jointly mediated by powder dosage form and small dose. Front. Pharmacol. 16. doi: 10.3389/fphar.2025.1679105. PMID: 41104334 PMC12521102

[B16] Wang F.-P. Li Z.-B. Che C.-T. (1998). Beiwudine, a norditerpenoid alkaloid from Aconitum kusnezoffii. J. Nat. Prod. 61, 1555–1556. doi: 10.1021/np960481t. PMID: 9868164

[B17] Wang T. Rangji C. Liu W. Ma J. Zhou R. Leng L. . (2024). Multi-omics on traditional medicinal plant of the genus Aconitum: Current progress and prospect. Molecules 30, 118. doi: 10.3390/molecules30010118. PMID: 39795175 PMC11722372

[B18] Wang T. Xu G. Liu Z. Ding X. Wang L. Leng L. . (2025). Integrated metabolite profiling and transcriptome analysis identify candidate genes involved in diterpenoid alkaloid biosynthesis in Aconitum pendulum. Front. Plant Sci. 16, 1547584. doi: 10.3389/fpls.2025.1547584. PMID: 40196428 PMC11973281

[B19] Wu L. Zhao B. Deng Z. Wang B. Yu Y. (2023). A biosynthetic network for protoberberine production in Coptis chinensis. Hortic. Res. 11, uhad259. doi: 10.1093/hr/uhad259. PMID: 38282690 PMC10812381

[B20] Xu D. Lin H. Tang Y. Huang L. Xu J. Nian S. . (2021). Integration of full-length transcriptomics and targeted metabolomics to identify benzylisoquinoline alkaloid biosynthetic genes in Corydalis yanhusuo. Hortic. Res. 8. doi: 10.1038/s41438-020-00450-6. PMID: 33423040 PMC7797006

[B21] Xu D. Wang Z. Zhuang W. Wang T. Xie Y. (2023). Family characteristics, phylogenetic reconstruction, and potential applications of the plant BAHD acyltransferase family. Front. Plant Sci. 14, 16. doi: 10.3389/fpls.2023.1218914. PMID: 37868312 PMC10585174

[B22] Yang D. Du X. Liang X. Han R. Liang Z. Liu Y. . (2012). Different roles of the mevalonate and methylerythritol phosphate pathways in cell growth and tanshinone production of Salvia miltiorrhiza hairy roots. PloS One 7, e46797. doi: 10.1371/journal.pone.0046797. PMID: 23209548 PMC3510226

[B23] Zhang R. Yang Q. Huang Z. Liu H. Gao J. Nie X. . (2026). A comprehensive review of the traditional usages, phytochemistry, pharmacology, toxicology, quality control and other applications of Aconitum kusnezoffii Reichb. J. Ethnopharmacol. 356, 120813. doi: 10.1016/j.jep.2025.120813. PMID: 41151648

[B24] Zhao D. Zhang Y. Ren H. Shi Y. Dong D. Li Z. . (2023). Multi‐omics analysis reveals the evolutionary origin of diterpenoid alkaloid biosynthesis pathways in Aconitum. J. Integr. Plant Biol. 65, 2320–2335. doi: 10.1111/jipb.13565. PMID: 37688324

